# Supplementary material for: Evaluating operational parameters of the careHPV, GeneXpert, AmpFire, and MA-6000 HPV systems for cervical precancer screening: Experience from Battor, Ghana
Source: PLOS Glob Public Health. 2023 Aug 25;3(8):e0001639. doi: 10.1371/journal.pgph.0001639 (PMC10456161; doi:10.1371/journal.pgph.0001639)
Supplement: Attachment — Submitted filename: Rebuttal_Four_Tests.docx [file pgph.0001639.s001.docx]

| **Manuscript number: PGPH-D-22-02127**  **Journal: *PLOS Global Public Health***  **Revised title: Evaluating operational parameters of the *care*HPV, GeneXpert, AmpFire, and MA-6000 HPV systems for cervical precancer screening: Experience from Battor, Ghana** | | |
| --- | --- | --- |
| **RESPONSES TO COMMENTS ON JOURNAL REQUIREMENTS** | | |
| **Reviewer’s comment** | **Authors’ response** | **Line numbers** |
| 1. We do not publish any copyright or trademark symbols that usually accompany proprietary names, eg (R), (C), or TM (e.g. next to drug or reagent names). Please remove all instances of trademark/copyright symbols throughout the text, including TM on References Section. | We have carefully removed all instances of (R), (C), and TM symbols next to proprietary names throughout the manuscript, including the references section. | - |
| 2. Some material included in your submission may be copyrighted. According to PLOS’s copyright policy, authors who use figures or other material (e.g., graphics, clipart, maps) from another author or copyright holder must demonstrate or obtain permission to publish this material under the Creative Commons Attribution 4.0 International (CC BY 4.0) License used by PLOS journals. Please closely review the details of PLOS’s copyright requirements here: PLOS Licenses and Copyright. If you need to request permissions from a copyright holder, you may use PLOS's Copyright Content Permission form. Please respond directly to this email or email the journal office and provide any known details concerning your material's license terms and permissions required for reuse, even if you have not yet obtained copyright permissions or are unsure of your material's copyright compatibility.  Potential Copyright Issues:  Figs 2 and 3: Please confirm (a) that you are the photographer; or (b) provide written permission from the photographer to publish the photo(s) under our CC-BY 4.0 license. | The images shown in Figs 2 and 3 were taken by the lead author during screening. They have neither been published elsewhere nor submitted to another journal. However, following the revisions and recommendations made by Reviewer 2, they have been removed. The manuscript currently contains no figures. | - |

| **RESPONSES TO REVIEWER 1** | | |
| --- | --- | --- |
| **Reviewer’s comment** | **Authors’ response** | **Line numbers** |
| This is a well-written and important contribution to the literature to inform HPV test choices for cervical precancer detection. It is very difficult to navigate the technical and practical differences in HPV test platforms, and this study provides a very helpful analysis in a real-world context in Ghana. My main comments relate to providing more information in the methods and results, and considering some additional points for discussion. | Thank you for your valuable comments on the practical relevance of our analysis in the Ghanaian context. Your comments and recommendations are highly appreciated. | - |
| Line 67: It would be helpful to add one more sentence here to provide a roadmap for the next four paragraphs (eg, the four HPV DNA tests that are considered in this study are __) | An extra sentence has been inserted, as suggested. | 67–71 |
| Line 85: With low-throughput machines, in the authors’ opinion, is it logistically feasible to add in HPV testing to existing TB program instruments? Xpert platforms in theory are great to accommodate multi-analyte detection, but I’ve run into issues where the machines are in use full time for TB and HIV testing, which seems to be the case for the authors too (line 300). It might be helpful to add a point into the discussion about whether this was the main factor that limited the overall use of GeneXpert in this study, or if another factor like cost was involved. | We agree with you that GeneXpert platforms, in theory, have the capability to facilitate multi-analyte detection. As you have highlighted, the operational demand for TB and HIV testing would likely occupy the machines extensively. Despite this competition, in our experience, limited use of the GeneXpert was not due to low throughput but due to cost. Women in Ghana have to pay from their pockets to access cervical precancer screening. Many could not afford the GeneXpert HPV DNA testing. The HPV test with the GeneXpert ran seamlessly with TB testing, the main reason the platform was placed in the hospital by the National Tuberculosis Programme.  We have highlighted this briefly in the discussion. | 344–347 |
| Line 92: I disagree that AmpFire is a simple process of sampling *for the user*, especially compared with GeneXpert. I do agree that AmpFire not requiring extraction (if samples are collected directly into Atila buffer) is simpler than requiring extraction like GeneXpert, but I think it’s important to clarify that “simpler” in this case refers to the molecular detection mechanisms, not the user steps. In practice, AmpFire requires a number of user steps that must be carried out by trained personnel to avoid environmental cross-contamination by amplicons due to its PCR plate format. GeneXpert, with minimal user steps that just require transferring sample to a cartridge with a transfer pipette, in my experience, is much simpler. | We have revised the sentence to avoid any confusion by highlighting that the AmpFire utilizes a simple molecular detection mechanism. The simplicity of GeneXpert has also been made clear. | 94–96 |
| Line 110: it would be helpful to define what is meant by ‘appropriateness’ earlier, rather than just in the discussion. I would suggest just replacing the term with throughput and sample batching, as defended in the discussion, for clarity. | We define ‘appropriateness’ as how suitable an HPV test platform is for implementation in various settings, encompassing the ability to meet the clinical needs of the screened population and adaptability to resource availability. Based on this and another comment made by Reviewer 2, we have included a subsection titled ‘Key definitions’ in the methods section in which we define appropriateness, ease of use, throughput, and diagnostic yield. We believe this would provide clarity to readers as to how these terms are used in the discussion. | 218–228 |
| Line 136: Throughout the whole “Laboratory processing of cervicovaginal samples for HPV DNA assays”, additional details are needed on sample collection. Different platforms are validated with different collection buffers—were all samples collected into ThinPrep? Were any samples converted from one buffer into another? How long were they stored for and under what storage conditions before testing? Were specimens self-collected or physician-collected, and using what sampling swabs/devices? What instruments were used for AmpFire and MA-6000 testing? Some of these are addressed in the discussion and Table 3 but should appear in the methods. | We did not convert any samples from one buffer to another. *care*HPV requires its own collection medium. For GeneXpert, we used PreservCyt (ThinPrep) which usually was a health worker collected sample (with a Cervex brush) that was rinsed in the ThinPrep. For AmpFire and MA-6000, we used both dry brush samples and ThinPrep, with the samples taken by health workers. Some women also preferred self-sampling (with the Evalyn brush). The self-samples were transported as dry brush samples to the laboratory. For health worker collected samples, the choice of whether dry brush samples or ThinPrep was used depended on what the women wanted after discussions with them. Women who wanted cytology in addition to HPV DNA testing had samples put in ThinPrep to be able to run both tests. Cytology could not be done on dry brush samples. Health workers used cytobrushes or cotton-tipped applicators depending on the availability. Cytobrushes were used more often. Samples for GeneXpert were usually run the same day. For AmpFire and MA 6000, the samples were usually run within two weeks. *care*HPV took much longer (sometimes up to 6 weeks) because samples needed to be batched. Dry brush samples were stored in a freezer at -16°C, while ThinPrep samples were stored in an airconditioned room at a temperature range of 16–20°C.  We have mentioned these details and the process of sample collection in a new subsection titled ‘Cervicovaginal sample collection and storage’ in the methods section. | 142–166 |
| Line 140: “lysate” should be “lysis buffer” I believe | This has been corrected. | 172 |
| Line 166: “lyse” should be “lysis” | This has been corrected. | 196–199 |
| Line 177: what is meant by “preprocessed”? Using what protocol? | Preprocessing for MA-6000 testing was performed by adding the manufacturer’s sample release reagent and incubating the mixture for 10 min at room temperature to isolate a pure fraction of DNA in solution. The sentence has been modified to incorporate this. | 207–210 |
| Line 203: Were demographics different between the women who were tested by each of the tests? | When disaggregated according to platform, we observed a similar age distribution among the four sub-cohorts of women tested across the platforms (*p*-value = 0.2481). On the other hand, the four platforms showed statistically significant differences in the distributions of religion (*p*-value <0.001), highest education level (*p*-value <0.001), whether or not a participant earned an income (*p*-value <0.001), number of children (*p*-value <0.001), smoking status (*p*-value <0.001), HIV status (*p*-value <0.001), and marital status (*p*-value <0.001) among women tested (Table 1).  These details have been highlighted in Table 1, which contains demographic characteristics of the women by platform used and in the results section. | Table 1  260–266 |
| Table 2: It would be helpful to see more detail in this analysis, and I suggest significantly expanding upon what is currently shown. Besides treatment, what were the percent of positives that correlated to cytologically negative. | Details of the histopathological diagnoses of the women included in this study have been partially described in a prior publication [1]. We believed describing the percentage of positives that correlated to cytologically negative results would not be beneficial for a number of reasons. The women were screened using different approaches. Of the 6056 participants, 1574 underwent standalone HPV testing, while 4482 underwent screening by way of concurrent HPV testing + a visual inspection method (3681 by visual inspection with acetic acid [VIA] and 801 with EVA mobile colposcopy). For each of these concurrently screened groups, treatment was offered to visual inspection ‘positive’ women, by way of LEEP or thermal coagulation, as per our institutional algorithm. Among 51 concurrently tested hr-HPV + and visual inspection ‘positive’ women, histopathology results were available for 12. The remaining women either opted for thermal coagulation or were managed conservatively (because they had minor lesions).  In light of these and other comments made by Reviewer 2 entreating us to clearly define the scope of our study to the operational parameters of the four tests, we have excluded all details pertaining to cohort treatment and histopathological results.  **Reference**  1. Effah K, Tekpor E, Wormenor CM, Amuah JE, Essel NO, Atuguba BH, et al. Concurrent HPV DNA testing and a visual inspection method for cervical precancer screening: A practical approach from Battor, Ghana. PLOS Global Public Health. 2023;3: e0001830. doi:10.1371/journal.pgph.0001830 | Table 2 |
| Line 285: Self-sampling has been shown to have higher cell counts, but slightly lower sensitivity than physician-sampling, which is contrasted with self-sampling increasing rates of HPV detection as proposed in the discussion. I would consider the possibility that higher rates of detection may reflect false positivity due to the PCR-plate format of the AmpFire and MA-6000 tests (I’ve seen very high levels of FPs on AmpFire in practice). Was quality control performed to monitor levels of false positivity? If not, I would include in the discussion that this was not evaluated but should be in the future to understand true sample differences versus platform differences. | Your hypothesis that the higher rates of detection might be due to false positives, especially considering the PCR-plate format of the AmpFire and MA-6000 tests, is indeed worth contemplating. At our laboratory, quality control measures are taken to mitigate this. The careHPV, AmpFire, and MA-6000 test kits come with positive and negative controls. Out of 96 wells, we run a maximum of 94 tests with at least one positive control and one negative control. Despite this, we believe it was still worth putting this forward in the discussion, as a potential area for future research in understanding true differences among samples versus those based on platforms. | 336–340 |
| Were any samples run on more than one platform? How did these compare? | While comparing the samples across multiple platforms would indeed provide valuable insights into consistency and agreement, this was not done in our study. The lack of testing on multiple platforms has been highlighted as a limitation. | 408–414 |
| Figure 1: This figure is hard to read with the background chosen. I would recommend cleaning this figure up a bit with a plain background, and if possible, more linear paths through the diagram. | In light of comments made by Reviewer 2 to carefully define the scope of our study to the operational parameters of the four tests, we have removed the original Figure 1 detailing our approach to patient triaging and treatment, as it was no longer relevant after revising the manuscript. | - |

| **RESPONSES TO REVIEWER 2** | | |
| --- | --- | --- |
| **Reviewer’s comment** | **Authors’ response** | **Line numbers** |
| Utilizing HPV DNA testing is recommended by the WHO for cervical cancer screening in middle- and low-income countries. Therefore, it is of interest to access and compare performances of various available HPV testing platforms that have been operated in low-resource settings, such as Ghana. The authors of this manuscript aimed to compare the performances of careHPV, GeneXpert, AmpFire, and MA-6000. Operational features (manufacturer, HPV types, sample type, batching, duration, throughput) were demonstrated for each platform. The authors also presented the number of tests performed, number of resulting HR-HPV positive tests and percentages using each platform, and treatments performed on women who had a positive test. | We thank Reviewer 2 for helping to enhance the depth and applicability of our article. Your input is highly appreciated. | - |
| There are several flaws in this study: |  |  |
| 1. The authors presented the HR-HPV positivity rates of each platform and attempted to compare the rates between different platforms. However, the rate calculation with respect to each platform was based on a specific cohort of women who received screening using a specific platform. Therefore, the differences in HR-HPV positivity rates between different platforms could potentially be attributed to the differences of the cohorts that the HPV tests were administrated on. The statistical tests and inference on positivity rates between different platforms are not meaningful in this case. Instead, the authors should compare and report the social, demographic, and primary screening details of the four cohorts of women who used different platforms. In addition, the authors should clearly state in the result section that the differences in HPV positivity rates between different platforms may be attributed to the differences of the cohorts. | We agree that differences in social, demographic, and screening history among the cohorts could potentially influence the positivity rates. To address this, we have revised our manuscript to include a detailed comparison of the demographic and clinical characteristics of the women in the four cohorts in Table 1 and in the results. Here, after stratifying the data by test platform, we used one-way ANOVA and the Kruskal–Wallis equality of proportions test to compare means and medians across the four cohorts and chi-squared tests to look for differences in the distributions of categorical characteristics among women screened with each test platform. We believe this will allow readers to better understand the context within which the hr-HPV positivity rates were obtained. | Table 1  236–240  260–266 |
| 2. Cytologic and histopathologic diagnoses of the study samples were mentioned in the introduction, however no actual data were reported in the results section. The correlations of HR-HPV testing results and cytologic and histopathologic results should be reported, which would provide some clinical evidence on accuracy of a specific HPV test. | Details of the histopathological diagnoses of the women included in this study have been partially described in a prior publication [1]. We believed describing the percentage of positives that correlated to cytologically negative results would not be beneficial for a number of reasons. The women were screened using different approaches. Of the 6056 participants, 1574 underwent standalone HPV testing, while 4482 underwent screening by way of concurrent HPV testing plus a visual inspection method (3681 by visual inspection with acetic acid [VIA] and 801 with EVA mobile colposcopy). For each of these concurrently screened groups, treatment was offered to visual inspection ‘positive’ women, by way of LEEP or thermal coagulation, as per our institutional algorithm. Among 51 concurrently tested hr-HPV + and visual inspection ‘positive’ women, histopathology results were available for 12. The remaining women either opted for thermal coagulation or were managed conservatively (because they had minor lesions).  In light of these and other recommendations entreating us to clearly define the scope of our study to the operational parameters of the four tests, we have excluded all details pertaining to cohort treatment and histopathological results.  **Reference**  1. Effah K, Tekpor E, Wormenor CM, Amuah JE, Essel NO, Atuguba BH, et al. Concurrent HPV DNA testing and a visual inspection method for cervical precancer screening: A practical approach from Battor, Ghana. PLOS Global Public Health. 2023;3: e0001830. doi:10.1371/journal.pgph.0001830 | Table 2 |
| 3. The authors presented treatment results and example images of women who underwent HPV testing using different platforms. However, these reports of treatment yield no relevance to the aim of this study and should be deleted from the manuscript. | We initially included these treatment results and representative images in an attempt to provide some context regarding the real-world outcomes of using the different testing platforms in combination with visual inspection methods. In agreement with your suggestion, we have removed all treatment results and colposcopic images, and concentrate on providing a more focused and coherent analysis of the operational aspects of the test platforms. | - |
| 4. The overall organization, clarity, English writing of the manuscript are suboptimal. | We appreciate your comments and would like to apologize for any issues related to the language quality of the original manuscript. After incorporating the suggested changes, the revised manuscript has been edited by a professional language editor and proofreader to ensure that it meets the journal’s required standard. | - |
|  |  |  |
| **Specific points that need to be addressed:** |  |  |
| **Title:**  • The tile of the manuscript is misleading, as it suggests that this is a study of actual screening for cervical cancer, which it is not. Nor is it a comparison of the four tests for cervical cancer screening, as no samples were tested by more than one of the assays. A more appropriate title would state that this is a description of the operative parameters of four HR-HPV tests among women living in Ghana. | We acknowledge the concerns raised and agree that the title should more accurately reflect the content and nature of the study. In response to your suggestion, we have modified the title to: “*Evaluating operational parameters of the careHPV, GeneXpert, AmpFire, and MA-6000 HPV systems for cervical precancer screening: Experience from Battor, Ghana*”. We believe this title accurately encompasses the focus of our study without implying a direct comparison or screening for cervical cancer. | 4–6 |
| **Abstract:**  • Line 43, The statement “the limitations of each test in terms of sensitivity and specificity depending on the local HPV genotype prevalence” is incorrect. Sensitivity and specificity are characteristics of a diagnostic test that are not dependent on prevalence of the disease or condition. In fact, sensitivity and specificity were not assessed for the four HPV testing platforms. Therefore, it should not be stated in the abstract. | We understand that sensitivity and specificity are inherent characteristics of diagnostic tests, and do not change with the prevalence of the condition. As we did not evaluate these, we have simplified the sentence to: “Factors worth considering are the financial implications of platform acquisition, costs to clients, and throughput when screening programs are not sufficiently large.” | 43–45 |
|  |  |  |
| **Introduction** |  |  |
| • There is no description of the Cervical Cancer Prevention and Training Center, Battor, where the HPV tests were conducted. | We have revised the subsection under the methods section, titled “Study design, setting, and data collection,” to provide a brief overview of the Cervical Cancer Prevention and Training Center, Battor. This includes information on its history, services offered, and its significance in cervical cancer prevention in Ghana and beyond, to help readers better understand the setting in which the study was conducted. | 121–129 |
| • End of Line 60 what are the aspects the authors refer to? How these aspects are related to the current manuscript. | In the original manuscript, “aspects” at the end of line 60 was used in reference to capturing factors associated with HPV infection, reasons for the existing low level of awareness about screening among women, suggestions to decentralize screening services, and debunking negative sociocultural and religious beliefs (as stated in the preceding sentence). As inserting this word could be confusing, we have deleted “in these aspects”, and revised the sentence to:  “For a country that has no clinical guidelines pertaining to cervical precancer screening and HPV management, it is essential to gather country-specific data to serve as a basis for policymaking and future research.” | 59–61 |
| • Line 71 Digene Hybrid Capture II technology needs reference here. | A reference has been inserted. | 74–75 |
| • Line 73 the authors mentioned the advantages of careHPV. What are the advantages that need to be stated clearly. | Here, we were referring to *care*HPV’s ease of implementation for screening, high user acceptance rate, and sustainability in terms of cost. To remove ambiguity, we have revised the sentence to:  “In addition to being easily implementable, economically sustainable, and having a high acceptance rate among users, there is little need for samples to be refrigerated and it can use a battery-operated machine [9].” | 74–79 |
| • Line 75 careHPV is adequately sensitive. What is the value? | Here, we cite the results of a meta-analysis of 4 studies that showed a pooled sensitivity of 92.3% (95% CI, 81.0–97.1). We have revised the sentence to:  “In a recent meta-analysis [11], *care*HPV showed adequate sensitivity (92.3%; 95% confidence interval [CI], 81.0–97.1) for detecting cervical intraepithelial neoplasia (CIN) grades 2+.” | 79–81 |
| • Line 89-90 how is use of GeneXpert related to reduction of loss of follow-up? | Ghana has no structured cervical precancer screening program and screening is largely opportunistic. Thus, we were of the view that because many rural hospitals on the National Tuberculosis Programme have the GeneXpert test platform, women could visit these centers for primary screening and follow-up if GeneXpert HPV tests become available at these centers. This would remove the extra cost involved in travelling to centers such as the CCPTC to get screened, possibly reducing loss-to-follow-up. After careful consideration, we have deleted that sentence as we believed the preceding sentence is clear enough. | 90–93 |
| • Line 110-111 the authors should give clear definitions for appropriateness, ease of use, throughput, and diagnostic yield, because they are the four endpoints of this manuscript. | We define ‘appropriateness’ subjectively as how suitable an HPV test platform is for implementation in a setting, encompassing the ability to meet the clinical needs of the screened population and adaptability to resource availability. We define ease of use as the simplicity and user-friendliness of the test platforms, including ease of sample collection, preparation, and handling, the complexity of the test procedure, and the need for specialized training. Throughput denotes the number of samples each platform can process within a given timeframe, as a measure of its capacity and efficiency, particularly in high-volume testing environments, while diagnostic yield refers to the ability of each HPV test platform to accurately identify hr-HPV in samples, as well as the ability to distinguish among types.  Because these were the four endpoints, we have provided a separate subsection titled ‘Key definitions’. We believe this would provide clarity to readers as to how these terms are used in the discussion. | 218–228 |
|  |  |  |
| **Materials and methods** |  |  |
| • A description of CCPTC should be provided. The authors should also provide a brief description of the population who attend CCPTC. | We have revised the subsection under the methods section, titled “Study design, setting, and data collection,” to provide a brief overview of the Cervical Cancer Prevention and Training Center, Battor. This includes information on its history, services offered, and its significance in cervical cancer prevention in Ghana and beyond, to help readers better understand the setting in which the study was conducted. | 121–129 |
| • Figure 1 presents the algorithm for screening and treatment in their institution, which is irrelevant to the aim of this manuscript. | We agree with this comment and have removed Figure 1. | - |
| • The authors should also describe the specific cohorts of women who received screening based on specific HPV test platforms. | The women were neither selected nor assigned to the different test platforms. Because each of the HPV DNA test platforms were introduced at different times at the CCPTC, the women generally had the option of choosing which test they wanted done, after giving them details, including how long it would take to get results and the cost involved. Some of the women who wanted their results the same day paid more for the GeneXpert instead of *care*HPV. In light of this, we have restricted our description to those based solely on the stratified analysis according to platform, rather than describing specifics of cohort selection. | 162–166 |
|  |  |  |
| **Results** |  |  |
| • Table 1 should be presented by specific cohorts of women who were screened using specific HPV testing platforms. | In addition to the sociodemographic and clinical characteristics shown in the original Table 1, we have stratified the data by test platform. Then we used one-way ANOVA and the Kruskal–Wallis equality of proportions test to compare means and medians across the four cohorts and chi-squared tests to look for differences in the distributions of categorical characteristics among women screened with each test platform. | 236–240  Table 1 |
| • Presentations of treatments are irrelevant to the aim of this study | In keeping with your recommendation to stick to the scope of the study, all treatments have been removed from the revised manuscript. | - |
| • Table 2 needs footnotes indicating HPV types tested in each assay, for example in careHPV, P3, P4, P5 of GeneXpert, etc. | Footnotes have been added to Table 2 showing the HPV genotypes identified by each test platform and their qualitative markers. | Table 2 |
| • Table 3 needs footnotes explaining 8h throughput of GeneXpert. | Footnotes have been inserted in Table 3 explaining the 8 h throughput of the GeneXpert platform and how it relates to the module and number of tests run. | Table 3 |
|  |  |  |
| **Discussion and Conclusion** |  |  |
| • This is a descriptive report of operational functions of the four HPV testing platforms. Because of the study design (each platform was performed among a specific cohort of women), no actual comparisons were made between the four platforms, except for descriptions of the functional features of each platform. Therefore, the authors should clearly state the scope of the study. | We acknowledge that our study design primarily focused on the operational functions of the four HPV testing platforms among different cohorts of women. Based on your suggestion, we have revised the manuscript to clearly state the scope of the study in both the introduction and discussion sections. | 106–112  290–294 |
| • The authors aimed to compare the four platforms in terms of appropriateness, ease of use, throughput, and diagnostic yield. The discussion section should be organized in discussions of the above features in separate paragraphs. | We understand that the discussion should ideally have been separated into paragraphs targeting each endpoint. However, in describing our experience, we found that these endpoints often have a bearing on one another. For instance, ease of use can affect throughput, and both can influence the appropriateness of a test platform in a setting depending on whether there is a high or low case load. Given these interdependencies, we believe that an integrated approach to the discussion was more suitable for providing a cohesive and comprehensive analysis. As such, we have structured the discussion section to present the information in a manner that reflects the relationships and interplay among these endpoints, rather than separating them into separate paragraphs. This, we feel, offers the reader a more realistic and practical understanding of the comparisons and the consequential considerations involved in selecting a platform for HPV testing. | - |
| • The authors should also provide a summary paragraph discussing advantages and disadvantages of each platform in low-resource setting use. In addition, the authors recommendation in use of different platform under various circumstances would be beneficial. | In response to this comment, we have provided a summary paragraph. Despite mentioning differences from the perspective of laboratory processing of specimen for each platform, we highlight the difficulty in comparing advantages and disadvantages of the platforms and making specific recommendations for low-resource settings in general because what works in one setting may not work in another. We thus summarize our successes and challenges under various circumstances (as per your recommendation), before segueing this with our conclusions. | 368–396 |
